# Cutaneous *Leishmania mexicana* in a child: A diagnostic challenge in rural Arizona

**DOI:** 10.1016/j.jdcr.2025.11.025

**Published:** 2025-11-24

**Authors:** Maria Epino, Christina Druskovich, Grace S. Saglimbeni, Harper Price, Danielle Vargas de Stefano

**Affiliations:** a Creighton University School of Medicine, Phoenix, Arizona; b Department of Internal Medicine, University of Utah, Salt Lake City, Utah; c Division of Dermatology, Phoenix Children's Hospital, Phoenix, Arizona; d Division of Pathology and Laboratory Medicine, Phoenix Children's Hospital, Phoenix, Arizona

**Keywords:** Arizona, biopsy diagnosis, cutaneous leishmania, infection, pediatric

## Introduction

Cutaneous leishmaniasis (CL) is a protozoan parasitic infection transmitted by female *Lutzomyia* sand flies representing the most common form of leishmaniasis worldwide. Although historically rare in the United States, recent reports demonstrate that *Leishmania mexicana* can sustain local transmission, particularly in Texas, Oklahoma, North Dakota, and Arizona.^1^, ^2^, ^3^, ^4^ In Arizona, semi-desert habitats provide suitable conditions for *Lutzomyia* sand flies, which can transmit *Leishmania* among mammalian reservoirs such as rodents and dogs.^3^

Recognizing pediatric CL is essential because it often presents as a persistent, painless ulcer that closely resembles common infectious or inflammatory dermatoses. When CL is not considered in children from affected U.S. states, diagnosis is frequently delayed, and patients undergo prolonged, ineffective courses of standard dermatologic treatments, as these ulcers typically respond only to Leishmania-specific therapy.^5^^,^^6^

We report a rare, biopsy-proven case of pediatric CL caused by *L. mexicana* infection, highlighting diagnostic challenges in non-endemic regions.

## Case presentation

**Fig 1:**
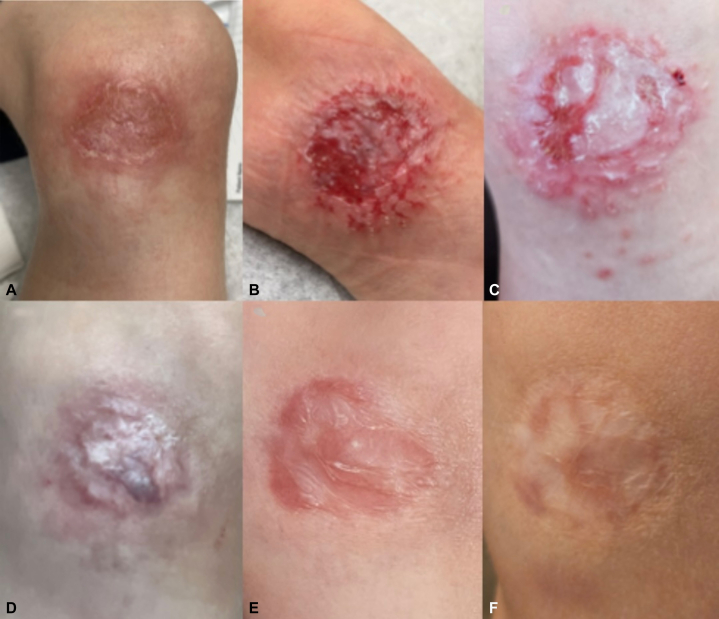
Clinical progression of a cutaneous *Leishmania mexicana* lesion on the left medial knee. **A,** Initial presentation with ulceration and surrounding erythema. **B,** Partial healing with reduced inflammation. **C,** Wound reopening with new papule formation. **D,** Stable healing with erythema from secondary trauma. **E,** Complete wound closure. **F,** Final image provided by family showing full resolution with atrophic scarring.

A 7-year-old boy with no significant past medical history presented with a 2.2 × 3.0 cm chronic, nonhealing ulcer on the left medial knee, present for 4 months (Fig 1, *A-F*). The lesion began as a small erosion, presumed secondary to minor trauma, according to his family. Over time, it developed into a shallow ulcer with firm, indurated borders and a pink-red base partially covered by fibrinous debris. Multiple topical and systemic antibiotics provided no improvement.

He was then referred to pediatric dermatology due to suspicion of contact dermatitis, which was not improving with topical medication. A detailed history revealed residence in rural Arizona with exposure to domestic animals, including cats, chickens, sheep, and cows, and a single trip to Mexico 9 months earlier. Due to chronicity, treatment resistance, and environmental exposures, a punch biopsy was performed for histopathology and tissue cultures. The clinical differential diagnosis was broad, including atypical infection or chronic inflammatory conditions like pyoderma gangrenosum.

**Fig 2:**
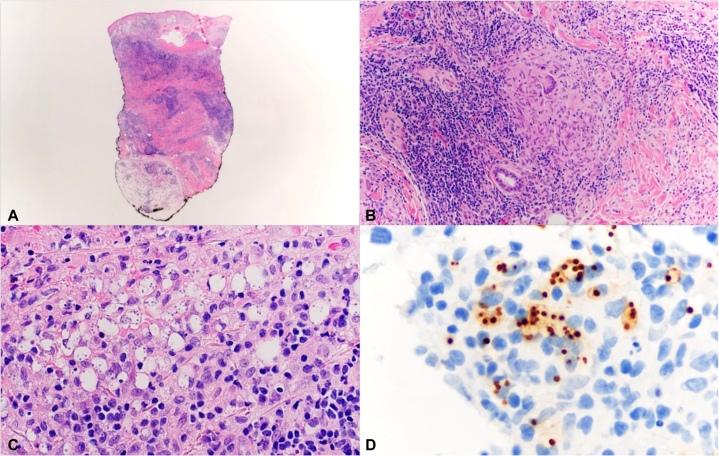
Histopathology of cutaneous *Leishmania mexicana*. **A,** Dense chronic granulomatous inflammation extending through the dermis into subcutaneous tissue (hematoxylin-eosin, low power). **B,** Well-formed epithelioid granuloma (hematoxylin-eosin, medium power). **C,** Numerous histiocytes containing intracellular vacuoles filled with amastigotes (hematoxylin-eosin, high power). **D,** Amastigotes stained strongly positive on CD1a immunohistochemistry.

Histopathology showed an extensive dermal infiltrate composed of histiocytes, lymphocytes, plasma cells, and neutrophils (Fig 2, *A*-*C*) . Numerous macrophages contained round to oval 2-4 μm organisms with eccentric nuclei, highlighted by Giemsa stain. Based on morphological findings and clinical presentation, the differential diagnosis included *Toxoplasma* sp., *Histoplasma* sp., and *Leishmania* sp. Fungal and Gram stains, PAS, GMS, AFB, and immunohistochemistry for *Toxoplasma* and *Histoplasma* were negative. Tissue cultures for bacteria, fungi, and atypical mycobacteria were also negative. CD1a staining was performed following Giemsa findings as an adjunct to enhance visualization of intracellular amastigotes within histiocytes. The suspicious microorganisms were strongly immunoreactive to CD1a. (Fig 2, *D*). Findings were consistent with *Leishmania* amastigotes. Additional speciation was performed and confirmed by the Centers for Disease Control and Prevention (CDC), which verified *L. mexicana*.

**Fig 3:**
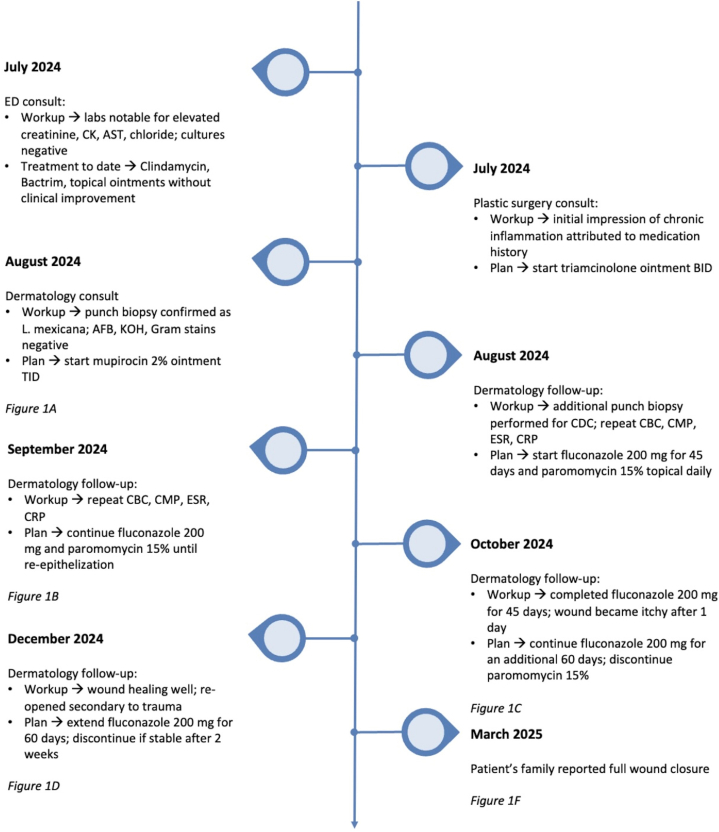
Clinical timeline of a pediatric patient with cutaneous *Leishmania mexicana*. The lesion was initially managed empirically with oral antibiotics and topical steroids without improvement. In August 2024, biopsy confirmed *L. mexicana*, prompting initiation of topical 15% paromomycin and oral fluconazole (200 mg daily). Serial laboratory monitoring was completed throughout treatment, and no abnormalities were observed. Gradual flattening and re-epithelialization were noted by October 2024, with nearly complete resolution by January 2025 and full wound closure by March 2025. The patient’s family remains in contact with the dermatology team, and complete wound closure has been maintained to date.

In consultation with our pediatric infectious disease team, the patient was initiated on 15% paromomycin and 200 mg of oral fluconazole daily for 6 weeks. Fluconazole was selected over other systemic agents such as itraconazole, miltefosine, and amphotericin B because of its favorable safety profile, oral bioavailability, and tolerability in pediatric patients. If fluconazole therapy failed or if clinical evidence of joint involvement developed, escalation to amphotericin B was planned. At 3-week follow-up, the wound showed decreased erythema and depth. Paromomycin was discontinued due to irritation and blister formation (Fig 1, *B*). Following the initial 45-day course of fluconazole, the wound improved but remained pruritic with new papule formation (Fig 1, *C*). The patient was prescribed an additional 60 days of fluconazole. On the next visit, the wound healed into an atrophic scar but reopened secondary to a new trauma from a bicycle fall (Fig 1, *D*). Despite this, the patient remained asymptomatic, without systemic symptoms or laboratory abnormalities. Due to the persistent wound, fluconazole was extended for an additional 60 days. Given the prolonged antifungal therapy, laboratory monitoring was conducted to assess potential side effects. The patient tolerated treatment well and completed approximately 5 months of fluconazole therapy, resulting in full wound closure and stabilization (Fig 1, *E* and *F*). A timeline summarizing the patient’s treatment course and clinical response is shown in Fig 3.

## Discussion

*Leishmania mexicana* is increasingly recognized as an emerging cause of CL in the United States.^7^ *L. ellisi* and *L. donovani complex* have also been reported in the U.S. as potential human-infecting species.^2^ Historically, most U.S. cases were linked to international travel; however, a multicenter review from Texas demonstrated that a substantial proportion is locally acquired, reflecting an evolving epidemiology.^1^ Furthermore, a 2025 nationwide review identified 89 documented autochthonous cases of CL reported since 1942, the majority from Texas, with 2 additional cases originating from Arizona.^2^ Limited reporting beyond Texas contributes to underrecognition, and climate change is predicted to promote the northward spread of leishmaniasis, a trend already observed within Texas counties.^2^ The evolving epidemiology of leishmaniasis should be carefully monitored, particularly as climate change, vector expansion, and limited case reporting continue to shape its emergence across the United States.

Currently, there is not a formal, universally adopted set of criteria from the World Health Organization defining autochthonous leishmaniasis, but in our patient, the long interval between travel and lesion onset, along with the absence of similar lesions among travel companions, highly suggested local acquisition. Additionally, with residence on a rural farm and frequent animal contact and outdoor exposure, the possibility of locally acquired infection becomes plausible given the documented presence of vector and reservoir species in the southwestern United States. In pediatric patients, chronic ulcers are often attributed to trauma, infection, or eczema, leading to diagnostic delays.^5^^,^^6^ In our patient, the lesion persisted for months despite multiple treatments before skin biopsy confirmed *L. mexicana*. Treatment options vary by species, geography, and availability, but our patient responded well to oral fluconazole, resulting in complete healing, supporting its safety as a pediatric option.^6^^,^^8^

Histopathology revealed a higher parasite burden consistent with *L. mexicana*, contrasting with the sparse granulomatous inflammation and necrosis of *L. braziliensis.* Although *L. mexicana* generally causes self-limited cutaneous ulcers, our patient developed an unusually deep and persistent lesion. This atypical course highlights that *L. mexicana* can occasionally behave more aggressively in pediatric hosts. CD1a immunohistochemistry was a useful adjunct by accentuating amastigotes within infected histiocytes. Although not specific for *Leishmania*, CD1a expression has been reported to aid in the morphological identification of infected cells.^9^ Several mechanisms have been proposed to explain this cross-reactivity, including antibody recognition and the potential transfer of CD1a molecules to the parasite during the exocytosis process.^9^ While these theories remain speculative, in the absence of species-specific immunostains, CD1a proved to be a valuable ancillary marker supporting Leishmania diagnoses.

## Teaching point

Like previously reported cases, our patient presented with a chronic, painless ulcer attributed to trauma or infection, leading to delayed biopsy and diagnosis. Prior pediatric reports from Texas describe similar diagnostic delays ranging from weeks to months, and lesions initially managed as bacterial or traumatic processes before *Leishmania* was identified.^10^ Early recognition of characteristic lesion morphology, particularly painless, indurated ulcers with raised borders, in an endemic region, should prompt histopathologic evaluation to prevent prolonged morbidity and incorrect management.^6^

Clinicians should maintain a high index of suspicion for *L. mexicana* in chronic, painless ulcers in patients from rural, animal-exposed regions of the U.S., where autochthonous CL has been reported, even in the absence of relevant travel history.

### Declaration of generative AI and AI-assisted technologies in the manuscript preparation process

During the preparation of this work the author(s) used ChatGPT in order to improve language. After using this tool/service, the author(s) reviewed and edited the content as needed and take(s) full responsibility for the content of the published article.

## Conflicts of interest

None disclosed.
